# Alterations in chromosome 1q in multiple myeloma randomized clinical trials: a systematic review

**DOI:** 10.1038/s41408-024-00985-0

**Published:** 2024-01-25

**Authors:** Karun Neupane, Gliceida Galarza Fortuna, Riyasha Dahal, Timothy Schmidt, Rafael Fonseca, Rajshekhar Chakraborty, Kelly Ann Koehn, Meera Mohan, Hira Mian, Luciano J. Costa, Douglas Sborov, Ghulam Rehman Mohyuddin

**Affiliations:** 1 Department of Internal Medicine, Albert Einstein College of Medicine/Jacobi Medical Center, Bronx, NY USA; 2 Division of Hematology and Hematological Malignancies, Huntsman Cancer Institute, University of Utah, Salt Lake City, UT USA; 3 Department of Internal Medicine, Universal College of Medical Sciences, Siddharthanagar, Nepal; 4 Department of Hematology-Oncology, University of Wisconsin, Madison, WI USA; 5 Department of Hematology-Oncology, Mayo Clinic, Phoenix, AZ USA; 6 Department of Hematology and Oncology, Columbia University Herbert Irving Comprehensive Cancer Center, New York, NY USA; 7 Department of Hematology and Oncology, Chub O’Reilly Cancer Center, Springfield, MO USA; 8 Department of Hematology and Oncology, Medical College of Wisconsin, Milwaukee, WI US; 9 Department of Hematology and Oncology, McMaster University, Hamilton, ON Canada; 10 Department of Hematology and Oncology, O’Neal Cancer Center, University of Alabama, Birmingham, AL USA

**Keywords:** Cancer epigenetics, Oncogenes

## Abstract

Extra copies of chromosome 1q21 (+1q: gain = 3 copies, amp >= 4 copies) are associated with worse outcomes in multiple myeloma (MM). This systematic review assesses the current reporting trends of +1q, the efficacy of existing regimens on +1q, and its prognostic implications in MM randomized controlled trials (RCTs). Pubmed, Embase and Cochrane Registry of RCTs were searched from January 2012 to December 2022. Only MM RCTs were included. A total of 124 RCTs were included, of which 29 (23%) studies reported on +1q. Among them, 10% defined thresholds for +1q, 14% reported survival data separately for gain and amp, and 79% considered +1q a high-risk cytogenetic abnormality. Amongst RCTs that met the primary endpoint showing improvement in progression free survival (PFS), lenalidomide maintenance (Myeloma XI), selinexor (BOSTON), and isatuximab (IKEMA and ICARIA) were shown to improve PFS for patients with evidence of +1q. Some additional RCT’s such as Myeloma XI+ (carfilzomib), ELOQUENT-3 (elotuzumab), and HOVON-65/GMMG-HD4 (bortezomib) met their endpoint showing improvement in PFS and also showed improvement in PFS in the +1q cohort, although the confidence interval crossed 1. All six studies that reported HR for +1q patients vs. without (across both arms) showed worse OS and PFS for +1q. There is considerable heterogeneity in the reporting of +1q. All interventions that have shown to be successful in RCTs and have clearly reported on the +1q subgroup have shown concordant direction of results and benefit of the applied intervention. A more standardized approach to reporting this abnormality is needed.

## Introduction

Despite advances in diagnostics and therapeutics, multiple myeloma (MM) remains associated with significant morbidity and mortality, with a broad range of patient survival observed. The cytogenetic alterations historically associated with high-risk disease include translocations (4;14), (14;16), (14;20), and deletion of chromosome 17p [1]. Recently, copy number gains of the long arm of chromosome 1 (+1q) have also been associated with worse outcomes [2].

Generally, +1q signifies the presence of one or more additional copies of a segment of chromosome 1q within the malignant plasma cells. Amongst MM with +1q, gain(1q) refers to those who possess only one extra copy of 1q, resulting in three total copies, while amp(1q) denotes patients exhibiting amplification of 1q, characterized by the presence of two or more additional copies, totaling four or more copies [2]. Through fluorescent in situ hybridization (FISH), around 32–40% of newly diagnosed MM is found to harbor +1q [3, 4]. A significant obstacle to understanding the role of +1q in MM is the need for more consistency in reporting and annotating cytogenetics [5]. However, the adverse prognostic implications of +1q have led to a clearer understanding of “double-hit” and “triplet-hit”, where an additive adverse prognostic effect of +1q is seen when combined with other high-risk abnormalities [6]. +1q has also been added to more recent myeloma staging systems, highlighting its prognostic significance [7, 8].

The introduction of immunomodulatory drugs (IMiDs), proteasome inhibitors (PIs), and anti-CD38 antibodies have significantly enhanced outcomes for patients with newly diagnosed MM. However, approximately 10–20% of patients have a 2-year PFS of ≤50% despite current treatment, which is categorized as high-risk MM [9–11]. Prior studies have reported amp(1q) to be linked to poor survival, which warrants recognition as a high-risk cytogenetic abnormality. Despite many studies indicating that gain(1q) is also high-risk, its influence seems less deleterious than amp(1q), though patients with gain(1q) still experience worse outcomes than those without it [2, 4, 12–15]. Moreover, the concomitant presence of other high-risk cytogenetic abnormalities or gene expression profiles could play a crucial role in determining the extent of additional risk conferred by this abnormality [2]. As an example, patients with both a high-risk status as determined by gene expression profiling (GEP70) and the presence of baseline gain(1q) had an especially poor response to daratumumab-based therapy [16].

To evaluate the current reporting trends of +1q in MM RCTs, we aimed to estimate the prevalence of +1q reporting, its impact on prognosis, how treatments impact outcomes for this subset of patients, and other characteristics of how it is reported through a systematic review of MM RCTs.

## Methods

Direct patient information was not obtained, and the data was gathered from publicly available and deidentified sources; therefore, this study was considered exempt from approval of the institutional review board.

A previously published systematic review and search strategy was utilized for this study [17]. We performed a search of three databases: (MEDLINE/PubMed, Embase, and Cochrane Registry of RCTs). The search terms used are highlighted in Supplementary Table 1. Two independent reviewers (KAK and GRM) screened all studies, and any conflict was resolved through mutual discussion. This systematic review was performed according to the Preferred Reporting Items for Systematic Reviews and Meta-Analyses (PRISMA) recommendations. Our search strategy was restricted to MM RCTs published in peer-reviewed journals from January 2012 to December 2022. This time period was chosen to ascertain the presumed increased reporting of +1q over time. All other studies were excluded, including editorials, case reports, case series, review articles, case-control, retrospective/prospective cohort, and single-arm studies. The search strategy was not restricted to language. Abstracts from conference proceedings that were captured on these databases via our search strategy, such as those on Embase, were included for final analysis in our study. This study was not registered on PROSPERO. Two authors (KN and GGF) performed and verified all data extraction. Extracted data was tabulated using Microsoft Excel (Microsoft, Redmond, Washington, United States). We identified the following characteristics of studies: name of the RCT, year of publication, number of participants, location of study (enrollment in one country versus multi-national), therapeutic agent under study, whether +1q was reported or not as a high-risk cytogenetic alteration, definition of +1q with respect to the percentage of cells with this abnormality detected, documentation of distinction between gain(1q) and amp(1q) in analysis, the prevalence of +1q in enrolled population, and the outcomes of patients [overall survival (OS) and progression-free survival (PFS)] in patients with +1q in the experimental versus control arm and in patients with or without +1q.

The primary outcome of this study was to determine the prevalence of +1q in MM RCTs. A key secondary outcome was to understand the prognostic significance of +1q in RCTs.

## Results

**Fig. 1:**
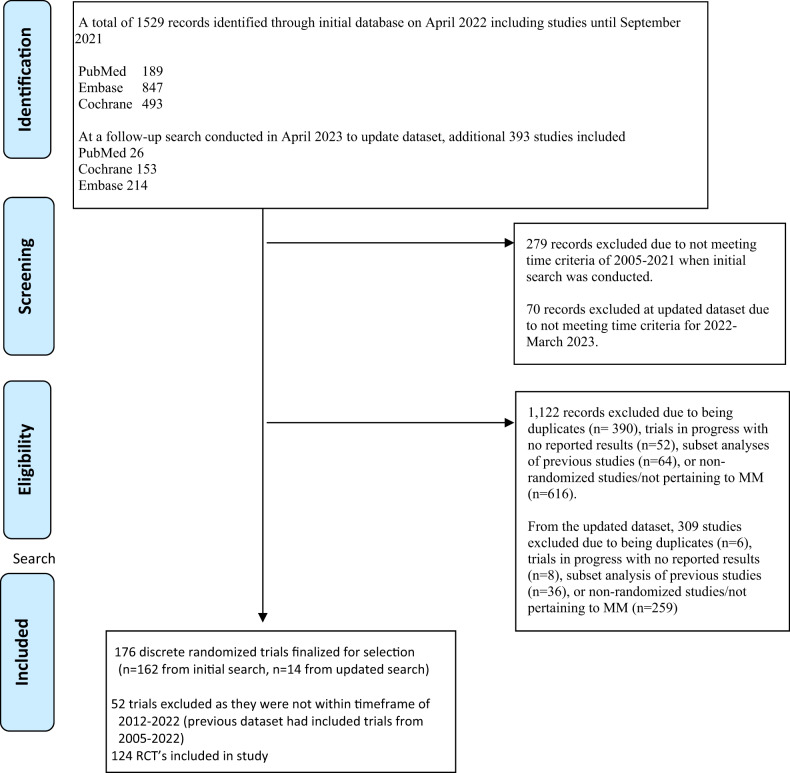
Flow diagram depicting our search strategy and study inclusion.

After excluding duplicate trials and trials that did not meet the inclusion criteria above, 124 discrete RCTs were identified (Fig. 1 highlights the study selection strategy). Table 1 highlights the characteristics of the included studies.

**Table 1:** Characteristics of multiple myeloma randomized clinical trials that reported +1q.

Study Characteristics	No. of studies reporting +1q (%)	No. of studies not reporting +1q (%)
Frontline or consolidation/maintenance	20 (71)	60 (63)
Relapsed/refractory	8 (29)	36 (37)
Multinational	12 (43)	53 (55)
Limited to a single country except the US	12 (43)	25 (26)
Limited to the US	4 (14)	18 (19)
Year 2012–2015	4 (14)	37 (39)
Year 2016–2019	11 (38)	49 (51)
Year 2020–2022	14 (48)	10 (10)

### Reporting of +1q

**Fig. 2:**
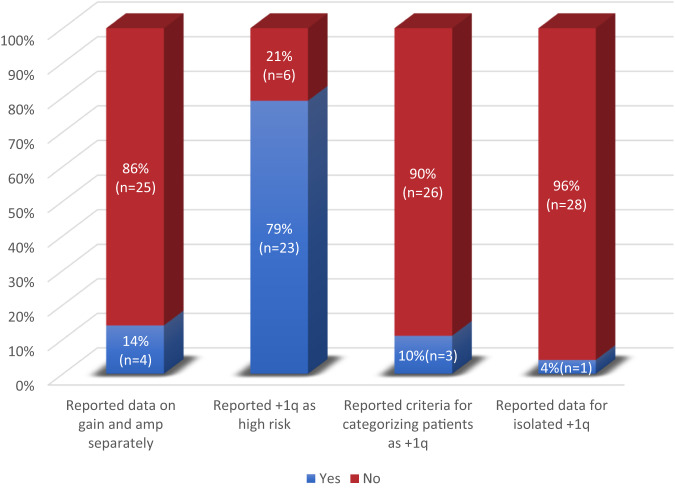
Bar graph depicting percentages of multiple myeloma trials reporting on +1q.

Among these trials, 29 (23%) studies reported data on +1q, including 26 studies that reported data in the primary manuscript and three studies that reported in separate publications. These RCTs reported 2754 patients with +1q representing 25% of all enrolled patients. Out of 29 RCTs, three trials (10%) specified the criteria for categorizing patients as +1q (for example, in IKEMA and IFM-99: the presence of at least three copies in at least 30% of analyzed plasma cells was required, and in ELOQUENT-2 positivity for 1q was assigned based on identifying at least one abnormal cell) [18–20]. Only four trials (14%) reported survival data on gain and amp separately [21–24], and the remaining 25 (86%) studies reported for gain or did not specify gain versus amp. One study reported three or more copies as amp(1q) in its original publication, although it did report separately for four or more copies in a follow-up publication [21, 25]. Only one (4%) RCT reported survival outcomes for patients with isolated +1q but no other high-risk cytogenetics [26]. Among the RCTs that reported +1q, 23 (79%) considered this a high-risk cytogenetic abnormality, with the remaining studies reporting on +1q, but not including it within the high-risk category. Reporting of +1q is summarized in Fig. 2.

Reporting of +1q has been increasing recently, with +1q being reported by 58.3% of MM RCTs published between 2020–2022 vs. 18.3% of MM RCTs published between 2016–2019 and 9.8% of MM RCTs published between 2012–2015. Most of these studies (86%) were from outside the US, and 71% included frontline or consolidation/maintenance therapy (Table 1).

### Survival outcomes

Amongst RCTs that met their primary endpoint showing improvement in PFS and clearly reported on +1q, the following drugs/regimens also improved PFS for those with +1q (when comparing HR for intervention versus control arm in the +1q subgroup): selinexor in BOSTON (HR 0.63, 95% CI 0.34–1.17, *p* = 0.07) [25], lenalidomide (len) maintenance in Myeloma XI (HR 1.5, 95% CI 0.9–2.7, *p* = 0.02) [26], isatuximab in IKEMA (HR 0.582, 95% CI 0.368–0.932) [18] and ICARIA (HR 0.41, 95% CI 0.2–0.7, *p* = 0.137) [27]. Although len maintenance improved PFS after autologous transplant as maintenance in Myeloma XI for patients with +1q overall (including those with other concurrent high-risk abnormalities), it did not appear to improve PFS for patients with isolated +1q (with no other concurrent genetic abnormalities) [26].

Several RCTs met their endpoint and showed improvement in PFS in the +1q cohort in the same direction as the overall study results but did not reach statistical significance. These included carfilzomib, len, dex, and cyclophosphamide vs. len, dexamethasone (dex), and cyclophosphamide (or thalidomide, dex, and cyclophosphamide in Myeloma XI+ (HR 0.63, 95% CI 0.38–1.06, *p* = 0.89) [28], the addition of elotuzumab to pomalidomide and dex (HR 0.56, 95% CI = 0.29–1.09) in ELOQUENT-3 [29], and bortezomib-based treatment before and after autologous stem cell transplantation vs. no bortezomib (HR 0.76, 95% CI 0.48–1.18, *p* = 0.22) in HOVON-65/GMMG-HD4 [23]. Although not powered for PFS, the addition of daratumumab to len and dex (HR 0.42, 95% CI 0.14–1.27) in GRIFFIN demonstrated a trend towards improvement in PFS for the +1q cohort [30].

Six RCTs, including ENDURANCE [22], IFM-99 [19], HOVON87/NMSG18 [31], HOVON-65/GMMG-HD4 [23], FORTE [4], and Myeloma IX [32] reported HR for patients with +1q in the trial (across both arms) compared to those without. Worse outcomes were seen in PFS and OS for those with +1q versus those without +1q in all these studies (Table 2). FORTE reported worse OS and PFS in patients with amp(1q) compared to gain(1q) (OS: HR 3.13, 95% CI 1.73–5.68, *p* < 0.001, and PFS: HR 1.84, 95% CI 1.21–2.81, *p* = 0.004) [4].

**Table 2:** Survival Outcomes in trials that reported separately on +1q.

Study name	Drug regimen	HR for OS (95% CI) *p*-value	HR for PFS (95% CI) *p*-value
**Hazard Ratio of Intervention vs control in patients with** +**1q**			
BOSTON [21, 25]	Sel, bort, and dex vs bort and dex	Amp: 0.85 (0.41–1.76) 0.33 Gain: 0.62 (0.40–0.96) nr	Amp: 0.63 (0.34–1.17) 0.07
Myeloma XI [26, 50]	Len maintenance	—	For all gain1q, 0.46 (0.33–0.62) 0.46 For isolated gain1q, 1.50, (0.90–2.70) 0.20
IKEMA [18]	Isa plus car–dex vs car–dex	0.57 (0.33–0.98)	0.58 (0.37–0.93)
Myeloma XI+ [28]	Cyc/thal, dex or cyc/len/dex vs cyc, car, len, dex	—	0.63 (0.38–1.06) 0.89
ENDURANCE [22, 34]	Addition of car vs bort to len and dex	Gain: 0.50 (0.28–0.90) 0.02 Amp: 1.56 (0.64–3.78) 0.32	Gain: 0.75 (0.49–1.14) 0.17 Amp:1.46 (0.73–2.92) 0.28
Myeloma XI [33]	Addition of vorinostat to len maintenance	1.04 (0.52–2.04) 0.45	1.2 (0.68–2.11) 0.45
ELOQUENT-3 [29]	Addition of elotuzumab to pom and dex	—	0.56 (0.29–1.09)
HOVON-65/GMMG-HD4 [23]	Bort before and after ASCT vs standard treatment without bort	0.58 (0.30–1.12) 0.1	0.76 (0.48–1.18) 0.22
ICARIA [27]	Isa plus pom and low-dose dex vs pom and low-dose dex	0.72 (0.48–1.07) 0.25	0.41 (0.2–0.7) 0.14
GRIFFIN [30]	Dara plus len and dex vs len and dex	—	0.42 (0.14–1.27)
SWOG-1211 [11]	Bort, len, and dex without or with elotuzumab	0.78 (0.39, 1.55)^a^	0.76 (0.46, 1.26)^a^
**Hazard ratio of patients with** + **1q (in all arms of trial) vs no** + **1q**			
HOVON87/NMSG18 [31]	Mel, pred, and len/thal	1.63 (1.13–2.35) 0.01	1.42 (1.10–1.83) < 0.01
ENDURANCE [22, 34]	Car/bort with len and dex	Gain: 1.40 (nr) 0.13 Amp: 1.78 (nr) 0.02	Gain: 1.46 (nr) < 0.01 Amp: 1.80 (nr) < 0.01
IFM-99 [19]	Thal maintenance	2.00 (1.56–2.58) < 0.01	1.42 (1.15–1.75) < 0.01
HOVON-65/GMMG-HD4 [23]	Bort before and after ASCT vs standard treatment without bort	Combined gain/amp: 1.90 (1.20–2.90) < 0.01 Gain: 1.66 (nr) < 0.01 Amp: 3.95 (nr) < 0.01	Combined gain/amp: 1.70 (1.30–2.30) < 0.01 Gain: 1.65 (nr) < 0.01 Amp: 2.48 (nr) < 0.01
FORTE [4]	Three arm trial respectively: first arm receiving car, len and dex with ASCT, second receiving car, cyc and dex with ASCT, and third receiving car-len-dex without ASCT. A second randomization then done for maintenance with car plus len or len alone	Gain: 1.88 (0.98–3.58) 0.06 Amp: 5.88 (3.1–11.17) < 0.01	Gain :1.65 (1.14–2.37) < 0.01 Amp: 3.04 (1.99–4.65) < 0.01
Myeloma IX [51]	Cyc, vincristine, dox and dex or cyc, thal and dex, followed by mel with ASCT vs either mel and pred or cyc, thal and dex	1.53 (1.20–1.94) < 0.01	1.46 (1.21–1.76) < 0.01

*HR* hazard ratio, *OS* overall survival, *PFS* progression free survival, *CI* confidence interval, *sel* selinexor, *bort* bortezomib, *dex* dexamethasone, *len* lenalidomide, *car* carfilzomib, *Isa* isatuximab, *cyc* cyclophosphamide, *vin* vincristine, *dox* doxorubicin, *thal* thalidomide, pom pomalidomide, *ASCT* autologous stem cell transplantation, *pred* prednisone, *mel* melphalan, *nr* not reported.

^a^Control vs intervention.

Among the RCTs that reported PFS and OS for the +1q group, three trials, SWOG-1211 [11], Myeloma XI [33] and, ENDURANCE [22] did not meet their primary endpoints. In Myeloma XI [33], no benefit was seen in the +1q group with the addition of vorinostat, and in SWOG-1211 [11], there was no statistically significant difference in PFS or OS in patients with +1q in patients treated with bort, len, and dex with or without elotuzumab. Though primary endpoint was not reached in ENDURANCE trial, the addition of carfilzomib vs. bortezomib to len and dex showed benefit in gain(1q) with HR 0.75, 95% CI 0.49–1.14, *p* = 0.17 but not in amp(1q) with HR 1.46, 95% CI 0.73–2.92, *p* = 0.281 [34].

### The gap in the evidence

Important interventions for which subgroup analysis of +1q was not presented in trial results, and hence conclusions about the efficacy of the drugs specifically for patients with +1q cannot be ascertained, include pomalidomide and ixazomib. Although GRIFFIN (a Phase 2) study has shown benefit of daratumumab in patients with +1q, other publications of daratumumab randomized phase 3 trials have not reported outcomes of the effect of daratumumab on +1q [30]. Two recent contemporary RCTs that isolated the effect of autologous transplant (DETERMINATION and IFM-2009) did not report +1q [35, 36]. However, in the FORTE trial, the adverse prognostic implications of +1q were not seen in the arm receiving carfilzomib, len, dex, and autologous transplant, indicating a possible role of carfilzomib and autologous transplant in ameliorating the adverse prognostic implications of +1q [24]. We did not find any RCT that enrolled patients exclusively with +1q.

## Discussion

In this systematic review of MM RCTs looking at +1q, we find three key findings. Firstly, in all studies that report outcomes in patients with +1q compared to those without, outcomes are poorer in those with +1q. Secondly, all interventions that have shown to be successful in RCTs also demonstrate benefit in those with +1q, with no specific evidence of any one particular therapy being uniquely effective in +1q. Third, we find considerable heterogeneity in the reporting of +1q in the literature and +1q to be inconsistently classified as a poor prognostic factor in subgroup analysis of randomized MM RCTs. Although various other narrative reviews have been conducted on this topic, our systematic review is the first attempt at categorizing reporting of +1q in all MM RCTs, through a systematic search [1, 2, 37, 38], and the knowledge gained from our review can serve as an impetus to standardize reporting in future RCTs.

Our results show that for interventions that were successful in meeting their endpoint in RCTs, the benefit was also seen in the +1q group. Conversely, for RCTs that were negative and did not meet their endpoint, no benefit was seen in the +1q group either for the intervention [33]. Given that such a substantial fraction of patients with MM have +1q, it is only logical that interventions that generally benefit patients with MM also benefit patients with +1q. It is claimed that drugs such as isatuximab may be uniquely efficacious in patients with +1q [39]. Whereas data on the efficacy of daratumumab specific for +1q was not reported in its trials other than the GRIFFIN trial, our overall results indicate that a drug that is generally effective in MM (such as daratumumab), is also likely to be effective in +1q. Further reporting on +1q in trials that have evaluated daratumumab in a randomized fashion would help clarify this. Targeted therapies specific to this subgroup could be an area of further interest, as we found no RCTs exclusively enrolling patients with +1q.

Most RCTs reviewed did not specify the percent of cells needed to harbor +1q to be classified as such. A prospective, non-randomized clinical trial investigated the prognostic implications of the size of the clones of +1q. They divided the patients according to the percentage of monoclonal plasma cells with the mentioned cytogenic alteration (20–50% vs. >50%). This trial did not find a statistically significant difference in PFS or OS between these groups [40]. However, the discovery of a small percentage of cells carrying +1q needs to be interpreted cautiously. This could be due to two different reason. Firstly, this could be a result of test being performed in a non CD138 enriched sample leading to an erroneous result not reflective of a new clone in malignant plasma cells. Secondly, this could represent the emergence of a new non-dominant sub-clone, a development that may be of future clinical significance over time.

Furthermore, we found that +1q was inconsistently reported as a high-risk cytogenetic abnormality. Some trials may report a higher overall proportion of patients with high-risk disease by including all patients +1q [24], whereas others may have a lower proportion of high-risk disease by only including those with other high-risk abnormalities such as deletion 17p or t(4;14). This is particularly noteworthy when comparing outcomes for high-risk diseases across trials and would benefit from standardization.

Data from Emory in a large cohort of 1000 patients treated with triplet induction (bortezomib/lenalidomide/dexamethasone), autologous transplant, and maintenance showed no adverse impact of gain(1q) without concurrent high-risk cytogenetics, but an adverse impact of four or more copies, or when present in conjunction with other cytogenetic features [12]. However, more recent data indicates a more likely direct prognostic impact of gain(1q). This is highlighted by a patient-level meta-analysis of 2,596 patients from three trials (German-speaking Myeloma Multicenter Group (GMMG), GMMG MM5 trial, EudraCT 2010-019173-16) and the Myeloma XI trial) that showed adverse prognostic impact of gain(1q) as well, with no discernible difference from amp(1q) in terms of prognosis [14]. Another meta-analysis of patients from the Myeloma IX and the XI trials also showed similar results- that gain(1q) was significantly associated with shorter PFS and OS compared to normal 1q copy number status. Amp(1q) was also linked to shorter PFS and OS compared to normal copy numbers of 1q, but there was no significant difference compared to gain(1q) [41]. Thus, while amp(1q) has been consistently shown to have an adverse effect on the prognosis as evidenced by the statistically significant worse HR for patients with amp(1q) in ENDURANCE [22, 34], HOVON-65/GMMG-HD4 [23] and FORTE [4], the effect of gain(1q) on prognosis is not clear. Although previous data from Mayo Clinic did not show a difference in median OS in patients with or without +1q [42], recent data shows that the presence of +1q was associated with high tumor burden, an advanced stage of disease and decreased overall survival on a multivariate analysis [43]. A summary of the data to date increasingly demonstrates an adverse prognostic impact of +1q, although the signal on whether the number of abnormal copies of +1q has a further effect on prognosis remains controversial, given conflicting results from the studies mentioned above.

Our results show that most RCTs do not provide granular detail on patients with +1q. As the data indicates, which concurrent cytogenetic abnormalities exist with +1q has a great impact on prognosis. As an example, in an analysis of 737 patients from Spain, +1q by itself did not improve the predictive value of the Revised International Staging System. However, the co-existence of hyperdiploidy with +1q improved the prognosis of patients undergoing stem cell transplant (10-year OS of 62.5% versus 96%) [44].

We recognize that unlike current knowledge that allows us to propose suggestions for future reporting of +1q, authors in earlier trials lacked the benefit of hindsight when studying this cytogenetic abnormality. Early issues with reporting were anticipated, and the diverse reporting approaches we found on our review do not cast a critical light on the efforts of other authors. Based on the evolving knowledge on this topic, we provide recommendations to standardize reporting of +1q in clinical trials. Firstly, the demographic information should clearly document the percentage of patients for whom testing for +1q was performed, and for what proportion of those patients was +1q detected. A clear description of the copy numbers of the 1q chromosome and consistent usage of the words amp to denote 4 or more copies or gain to denote just three copies is paramount. Description of the concurrent cytogenetic abnormalities (hyperdiploidies, or high-risk features) and the proportion of patients should be clearly reported in the demographics table. These subgroups of patients (+1q overall, those with only three copies, those with 4 or more copies, those with isolated +1q with no other abnormalities, those with +1q and other hyperdiploidies, and those with +1q and other high-risk cytogenetic features) represent distinct biology, and pre-planned subgroup analysis of these patients should be reported, allowing future patient-level meta-analysis of granular data. Furthermore, clear definitions of what threshold of cells for which +1q is found will aid in consistent analysis in the future. A recent analysis of 2596 patients from the German group had a cut-off of 10%, which would be a reasonable threshold to utilize for future studies [14]. The HARMONY alliance from Europe, which recently contributed to the development of the Second Revision of the International Staging System (R-2 ISS) of Myeloma represents another pivotal step forward in further understanding the prognostic implications of +1q [7]. Given that new staging systems now incorporate +1q, implementation of these staging systems will lead to improved reporting and understanding of +1q. As our understanding of cytogenetic abnormalities evolves, it is increasingly clear that there is a cumulative risk imparted by having +1q in addition to other cytogenetic abnormalities, and it is encouraging to see recent trials such as the IsKia study of isatuximab, carfilzomib, lenalidomide, dexamethasone vs carfilzomib, lenalidomide, dexamethasone presented at the American Society of Hematology 2023 meeting clearly demarcate outcomes based on the number of high-risk cytogenetic features [45]. Such analysis have been performed for other studies done recently [46, 47].

Although our analysis focused on the prognostic implications of +1q and not deletion 1p, abnormalities of deletion 1p may co-exist with +1q, and may compound the inferior prognostic implications of +1q [48]. Furthermore, biallelic deletion of 1p has been shown to be a particularly poor prognostic factor, based on a retrospective study of 2551 patients [49]. It is crucial that these cytogenetic abnormalities be clearly listed in future studies, and we believe that these abnormalities should be demarcated and their prognostic impact isolated rather than lumped as “chromosome 1 abnormalities”.

Limitations of our study include that some relevant or recent studies may not have been picked up despite searching three datasets. Furthermore, our study also carries the limitations inherited with systemic reviews, such as publication bias (studies with positive results are more likely to be published compared to those with negative results), selection bias, and selective outcome reporting bias.

## Conclusion

This systematic review finds significant heterogeneity in +1q reporting in recent RCTs, and an association of +1q with poor outcomes when found. We find that interventions that generally work for patients with MM, also demonstrate efficacy in the +1q subgroup. We propose standardization of +1q reporting to better understand the implications of this abnormality.

### Supplementary information

Supplementary Table 1: Search terms used
