# Supplementary material for: Alterations in chromosome 1q in multiple myeloma randomized clinical trials: a systematic review
Source: Blood Cancer J. 2024 Jan 25;14(1):20. doi: 10.1038/s41408-024-00985-0 (PMC10810902; doi:10.1038/s41408-024-00985-0)
Supplement: Supplementary file 1 — Supplementary Table 1: Search terms used [file 41408_2024_985_MOESM1_ESM.docx]

Supplementary Table 1: Search terms used

| SN | Search terms |
| --- | --- |
| 1 | Myeloma OR myelomas OR Myelomatos* OR Kahler-Disease |
| 2 | "Multiple Myeloma"[Mesh] |
| 3 | "Clinical Trial, Phase III" [Publication Type] |
| 4 | "Clinical Trials, Phase III as Topic"[Mesh] |
| 5 | (Randomized controlled trial[pt] OR controlled clinical trial[pt] OR randomized[tiab] OR placebo[tiab] OR drug therapy[sh] OR randomly[tiab] OR trial[tiab] OR groups[tiab] NOT (animals [mh] NOT humans [mh])) |
| 6 | 'myeloma'/exp |
| 7 | 'phase 3 clinical trial'/exp |
| 8 | 'phase 3 clinical trial (topic)'/exp |
| 9 | 'Crossover procedure':de OR 'double-blind procedure':de OR 'randomized controlled trial':de OR ‘single-blind procedure':de OR (random* OR factorial* OR crossover* OR cross NEXT/1 over* OR placebo* OR doubl* NEAR/1 blind* OR singl* NEAR/1 blind* OR assign* OR allocat* OR volunteer*):de,ab,ti |
